# Spoken Word Recognition Errors in Speech Audiometry: A Measure of Hearing Performance?

**DOI:** 10.1155/2015/932519

**Published:** 2015-10-18

**Authors:** Martine Coene, Anneke van der Lee, Paul J. Govaerts

**Affiliations:** ^1^Department of Language, Literature and Communication, Language and Hearing Center Amsterdam, VU Free University Amsterdam, De Boelelaan 1105, 1081 HV Amsterdam, Netherlands; ^2^The Eargroup, Herentalsebaan 75, 2100 Antwerpen, Belgium

## Abstract

This report provides a detailed analysis of incorrect responses from an open-set spoken word-repetition task which is part of a Dutch speech audiometric test battery. Single-consonant confusions were analyzed from 230 normal hearing participants in terms of the probability of choice of a particular response on the basis of acoustic-phonetic, lexical, and frequency variables. The results indicate that consonant confusions are better predicted by lexical knowledge than by acoustic properties of the stimulus word. A detailed analysis of the transmission of phonetic features indicates that “voicing” is best preserved whereas “manner of articulation” yields most perception errors. As consonant confusion matrices are often used to determine the degree and type of a patient's hearing impairment, to predict a patient's gain in hearing performance with hearing devices and to optimize the device settings in view of maximum output, the observed findings are highly relevant for the audiological practice. Based on our findings, speech audiometric outcomes provide a combined auditory-linguistic profile of the patient. The use of confusion matrices might therefore not be the method best suited to measure hearing performance. Ideally, they should be complemented by other listening task types that are known to have less linguistic bias, such as phonemic discrimination.

## 1. Introduction

Speech perception refers to the mapping of acoustic and sometimes visual or haptic signals onto language forms [1]. It is part of the* speech chain* which includes the processes of speech production, transmission, and perception [2]. In this paper, we will be concerned with the latter process. Whenever a speaker utters a sentence, speech sound waves travel to the outer ear, middle ear, and cochlea and are transformed into neural activity which is received and decoded by the brain. This involves two types of hearing processes: the first one is based on sensory information obtained at the level of the ear itself, providing the necessary information in a* bottom-up* way; the second refers to the cognitive part of the perception process and brings in* top-down* information.

A particular way of capturing the proper functioning of the speech chain is by measuring input and output [3], that is, by comparing the speech stimulus as it was originally uttered by the speaker to the way it is understood by the listener in the so-called stimulus-repetition tasks. An implementation of this principle can be found in speech audiometry, a clinical examination that is commonly used to assess the impact of a potential hearing deficit on speech understanding.

In this study, we will investigate the performance of participants with normal hearing on a speech audiometric test battery. Conventional speech audiometric test settings involve the repetition of short words that are presented acoustically to the tested individual. The stimuli typically consist of monosyllabic CVC words. The use of this type of stimuli has the advantage of providing little linguistic context from which to derive information that has been missed. Hence, the results of the test are generally considered to be highly informative with respect to the bottom-up processing of speech information in hearing, that is, at the level of the inner ear.

Our main aim is to gain more insight into phoneme substitutions which are found in speech audiometric data obtained from listeners with Dutch as a native language. In the clinical audiological practice, error patterns in phoneme perception are often analyzed by means of a phoneme confusion matrix. The outcomes are interpreted in terms of the particular configuration of hearing loss of the patient [4]. Apparently, the underlying assumption to do so is that deficits in the auditory periphery are the most apparent source of speech perception difficulties.

The central question that is raised in this paper addresses this particular assumption: Is it indeed the case that the erroneous replacement of a missed consonant is best explained by peripheral influences? In other words, do listeners make maximal use of the auditory information that is accessible to them? Or do they rather fill in the gap based on linguistic information? This question is operationalized by means of three hypotheses which relate to the previously mentioned bottom-up and top-down processes that underlie successful speech understanding.

The remaining part of the paper is structured as follows. In Section 2, we will first discuss the auditory component of the phonemes by which the speech stimuli are built up by means of articulatory features and how they may influence word identification. In Section 3, we will discuss the influence of linguistic factors on the replacement of a missed phoneme by a particular alternative. The aim of the study, its methods, and materials are given in Sections 4 and 5, respectively. In Section 6, the results of the analyses are described and are further discussed in Section 7. Finally, the conclusions of our research are found in Section 8.

## 2. Auditory Component

As was previously mentioned, the acoustic stimuli that are typically used in speech audiometric test batteries consist of short words. These are combinations of phonemes, that is, small units of speech sound (consonants or vowels) that are capable of conveying a distinction in meaning. A particular way to describe the phonetic content of the phonemes by which the stimuli are made up is by means of articulatory features. These speech features commonly involve voicing, nasality, affrication, duration, and place of articulation. Particular models of speech perception take listeners to be sensitive to these different phonetic features.

In information transfer (IT) analysis [5, 6], for instance, speech stimuli are compared to their repetitions in view of determining the fraction of the original information that has been transmitted to the listener for each feature independently. Information transfer is said to be complete if the listener does not make any confusions between phonemes belonging to a different articulatory feature category; for example, if a voiced bilabial /b/ is replaced by a voiced labiodental /d/, voicing is said to be transmitted whereas place of articulation is not. In case of random guessing and biased responses, input and output will be independent and the IT metric for a speech feature will yield a score of 0%. As such, the model measures the information encoded in the input and in the output and the covariance between them.

Under optimal listening conditions, listeners with normal hearing will rather easily obtain information transfer scores which are close to 100%; that is, they hardly make any errors when repeating the short CVC words that are presented to them. However, in difficult listening conditions, for example, when the stimulus word is presented in the presence of masking noise, the relative information transfer is significantly reduced. As expected, the portion of information which is transmitted drops as a function of the decreasing signal-to-noise ratio (SNR). Importantly, not all features are equally affected in their transmission: for example, whereas voicing and nasality are still discriminable at a SNR of −12 dB, the phoneme's place of articulation is hardly distinguishable even at a SNR of 6 dB [5]. By comparing the transmission rates of the articulatory features under similar listening conditions, it thus becomes possible to determine their relative prominence for a given population of listeners. For hearing impaired listeners, it has been shown that places of articulation errors are more prevalent followed by errors in the manner of articulation [7, 8].

## 3. Linguistic Component

There is an overwhelming body of literature reporting on nonauditory factors influencing speech understanding. It thus seems reasonable to expect that the potential variation in speech repetition errors cannot be fully explained in terms of the abovementioned articulatory features. Adequate processing of the perceived stimulus crucially relies on postcochlear processes as well, including key features of the central auditory system and the cortex, individual cognitive skills, and, most importantly, also information which comes from the linguistic system itself. In recent studies on speech perception, there is an important focus on nonauditory parameters, giving rise to a new interdisciplinary field of research (“cognitive hearing science”; see, e.g., [9]).

In healthy hearing adults, both auditory and linguistic factors may be taken to contribute to word identification accuracy. This is especially the case for stimuli presented in noise conditions. In individuals in which lower-level sensory processing is failing, top-down influences become proportionally more important: to fill in the missing gaps in the incoming speech signal, listeners can receive feedback from different levels of linguistic knowledge (see, e.g., [10–15] amongst many others).

Fortunately, language is an intrinsically redundant system in which a high amount of information that is relevant for speech understanding is represented more than once in the signal. This holds for different components of language, ranging from speech over morphology to complex syntax. Linguistic redundancy becomes particularly relevant when part of the acoustic information is missing. The* phonemic restoration effect* [16], for instance, is one of several phenomena proving that listeners are able to fill in missed phonemes based on nonauditory information.

For the past few decades, many scholars have investigated how listeners accomplish such a complex task. It is commonly believed that the appropriate comprehension of incoming speech is partially guided by the preceding linguistic context; that is, it enables listeners to make predictions with respect to how a sentence is likely to be continued. Evidence from (computational) psycholinguistic experiments indicates that words are identified more rapidly and more accurately when occurring in sentence contexts in which they have high probability to occur based on semantic and/or syntactic grounds [17–19].

In the literature, there is an ongoing debate with respect to the particular mechanisms underlying the potential serving role of linguistic context in speech comprehension. Within particular models of speech processing, it has been claimed that auditory processing, even at the level of early sensory analysis of the speech signal, is affected by top-down constraints from lexical and discourse processes [20–24]. Yet other, that is, modular, accounts rather follow the perspective of a* feed-forward* architecture, in which the output of early stage auditory processing module is passed on to the next interpretational level without feedback mechanisms that would allow the output of the first module to be corrected [25, 26].

In the context of the present study on speech understanding at the word level, two particular linguistic factors are of interest: (i) the* phonological neighborhood size* and (ii) the* frequency* of the word itself. With respect to the first factor, it is taken that the number of existing alternatives a given word has based on just one different phoneme can affect the listener's understanding of that word.* In concreto*, it takes more time to identify a word when several phonological neighbors are potential candidates. Part of the task of the listener is thus to eliminate the alternatives from his/her accessible lexical memory [27, 28]. Importantly, the impact of phonological neighborhood size on speech perception has also been attested in listeners with a hearing impairment [29].

Secondly, the distribution of a word in a given neighborhood may also be described in terms of frequency of usage [30, 31]. Most of the studies investigating the effect of word frequency on speech perception have found that there is a significant bias favoring the identification of words with a higher frequency of occurrence as compared to low-frequent words [32]. Again, this is the case both for listeners with normal hearing [27] and for listeners with a hearing impairment [29].

## 4. Aim, Research Questions, and Hypotheses

It has been well documented that there is no one-to-one relation between hearing performance based on pure-tone thresholds and speech understanding (see amongst others [33–35]). Sometimes listeners with relatively high pure-tone thresholds may perform unexpectedly well on word recognition tasks and vice versa. As oral communication is a social skill that heavily relies on the ability to hear and understand speech, in current clinical audiological practice, speech audiometry has become a fundamental tool in the assessment of hearing performance. In tandem with pure-tone audiometry, speech audiometric outcomes are taken to help the audiologist in determining the degree and type of hearing loss, to provide information regarding discomfort or tolerance to speech stimuli, and to measure an individual's functional hearing ability.

The information obtained from this complementary test procedure may also be used to predict a patient's gain in hearing performance with hearing devices and may help to optimize the device settings in view of maximum output.

Yet, an important drawback in the use of speech audiometric test batteries for hearing performance assessment is that current test batteries do not typically use stimuli that are controlled for both auditory and linguistic features. This implies that obtained word identification accuracy scores might not be unequivocally attributed to hearing performance. Against the background of the above sketched state of the art, the main aim of the present study is therefore to gain more insight into the proportional contribution of auditory versus linguistic factors in current speech audiometric word identification tasks.

In this paper, the central question that is raised is at the heart of the ongoing debate with respect to the interaction versus autonomous processing models in speech perception research: How do auditory cues, phonological neighborhood, and word frequency contribute to phoneme identification errors in word recognition tasks?

To answer this question, three hypotheses are raised in which particular predictions are made with respect to word repetition based on the three cues under investigation:

- The* auditory* hypothesis takes phoneme identification errors to be mainly driven by bottom-up (sensory) information alone: it predicts that the variance in erroneous responses to a given stimulus is best explained by the random choice out of the entire set of phonemes which is available in a given language, regardless of whether this yields an existing word or not.
- The* lexical* hypothesis predicts that erroneous responses will contain significantly more “existing” words than “nonsense” words.
- The* frequency* hypothesis takes the frequency usage of a word to be the most important factor explaining the variance in erroneous responses given by the listener.

## 5. Materials and Methods

### 5.1. Materials

The complete database on which the present analysis is built consists of 184 435 stimulus-response pairs of CVC words. These are drawn either from prerecorded wordlists that are commonly used in speech audiometric test batteries in the Dutch-speaking area (see, e.g., [36]) or from phonemically balanced lists obtained from daily readings of the participants themselves [38]. For an example of such CVC wordlist, see Table 4.

In agreement with a classical speech audiometric test procedure, the CVC words were presented acoustically to the participants, and their repetitions were recorded and subsequently scored on a phonemic level by an experienced audiologist. In line with clinical audiological standards, the presentation level of the stimuli ranged from 40 to 70 dB [39].

A total of 230 participants (146 men, 84 women) with normal hearing abilities participated in this study. They were listeners recruited from the Netherlands or Belgium (Flanders) having Dutch as a native language. Prior to participation, their speech production was judged according to the Speech Intelligibility Rate (SIR [40]). Participants whose SIR did not reach the level of* complete intelligibility* were excluded from the study. As can be read from Table 1, the obtained data from these participants contained over 21 000 uttered word repetitions of which 1957 stimulus-response pairs contained single phoneme errors.

### 5.2. Method

From the abovementioned database, all word types were selected which had at least 1% of consonant confusions. Based on this criterion, the database was narrowed down to word-initial and word-final consonant confusions; these were further analyzed in view of testing the abovementioned* auditory, lexical*, and* frequency* hypotheses.

With respect to the auditory hypothesis, each (erroneous) consonant response was analyzed in relation to its potential alternatives, the latter consisting of the set of consonants occurring in either word-initial or word-final position in the Dutch language. Specifically, within this hypothesis, is it predicted that in a stimulus such as Dutch /bEl/ “bell” the odds that the word-final consonant /l/ is replaced by (erroneous) /m/ (resulting in a “nonsense” word /bEm/) are the same as for any other phonemic alternative whether yielding “nonsense” words (/bEr/) or words that are part of the Dutch lexicon such as /bEn/ “am,” /bEs/ “berry,” and /bEk/ “beak.” Importantly, alternatives are defined based on the consonant inventory of the target language, regardless of whether a particular replacement will yield an existing word.

As for the* lexical hypothesis*, a similar comparison is made between the given response and its potential alternatives, but at the exclusion of nonexisting words in Dutch. Within this hypothesis, based on the above given stimulus /bEl/ “bell,” /bEr/ would not be considered as a potential alternative whereas /bEk/ “beak” would. The decision of whether a word is part of the Dutch lexicon has been defined based on* Van Dale's* explanatory dictionary of the Dutch language [41].

Finally, under the* frequency hypothesis,* it is expected that the frequency of usage of the potential response determines the choice of a particular consonant replacing the original one. In this study, word frequencies were calculated based on the Corpus of Spoken Dutch (Corpus Gesproken Nederlands [42]), a 9 000 000-word corpus of contemporary Dutch as spoken in the Netherlands and Flanders by adult-speakers. From this reference corpus, mispronunciations, foreign words, or uncertain pronunciations were excluded, but dialectal or regional pronunciations were not. With respect to the stimulus-response pair /bEl/-/bEm/, the frequency hypothesis would predict the listener to prefer /bEn/ “am” over other less frequent alternatives such as /bEK/ “beak.”

At a more detailed level, within each hypothesis, the obtained responses are compared to their potential alternatives in terms of categories of phonetic features such as “voicing,” “place,” and “manner of articulation” (see Table 5 for an overview, based on [37]). For each articulatory feature, the observed and expected values are calculated and standardized following

(1) $$\begin{array}{c} F=\sqrt{\frac{{\left(Fa_{\text{OBS}+\text{HE}}-Fa_{\text{EXP}}\left(Fa_{\text{OBS}+\text{HE}}+\cdots +Fn_{\text{OBS}}\right)\right)}^{2}+\cdots +{\left(Fn_{\text{OBS}}-Fn_{\text{EXP}}\left(Fa_{\text{OBS}+\text{HE}}+\cdots +Fn_{\text{OBS}}\right)\right)}^{2}}{{\left(Fa_{\text{OBS}+\text{HE}}+\cdots +Fn_{\text{OBS}}\right)}^{2}}}, \end{array}$$

where *Fa* = articulatory feature (voicing, place, or manner of articulation), *Fa*,…, *n* = feature value (e.g., voiced/voiceless), *Fa*_OBS_ = the observed value, *Fa*_EXP_ = the expected value, and HE = hypothetical error.

## 6. Results

Statistical analysis was done by means of an analysis of variance with the word-internal position of the target consonant (initial versus final) and its phonetic make-up in terms of articulatory features (voicing versus place versus manner) as independent factors and the results for the different consonant replacement strategies (auditory versus lexical versus frequency of usage) as dependent variables. The significance of the main and interaction effects was determined based on Greenhouse-Geisser or Huynh-Feldt corrected values [43]. Post hoc testing was done using the Bonferroni method. Effect-sizes have been determined based on the partial *η*².

**Figure 1:**
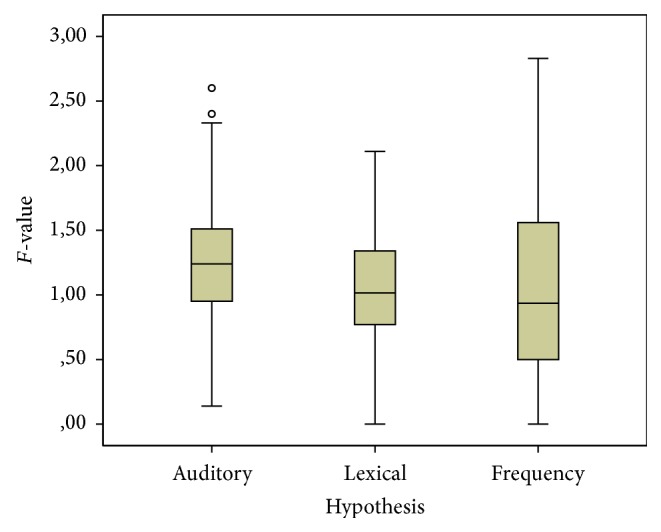
Standardized distances between observed and expected values for erroneous consonants replacements for auditory, lexical, and frequency routes in hearing listeners. Boxes: range between 25th and 75th percentile, whiskers: 1.5*∗*IQR, and central point: median. Circles: outliers (>1.5*∗*IQR).

**Figure 2:**
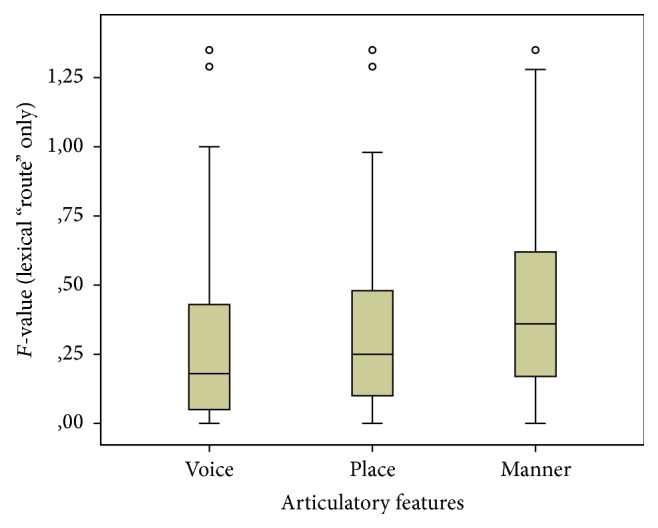
Standardized distances between observed and expected values for erroneous consonants replacements for the speech features “voicing,” “place,” and “manner” of articulation in hearing listeners. Boxes: range between 25th and 75th percentile, whiskers: 1.5*∗*IQR, and central point: median. Circles: outliers (values > 1.5*∗*IQR).

(1) The main effect of* replacement strategy* (i.e., the auditory, lexical, or frequency route) yielded an *F* ratio of *F*(1.288,171.34) = 11.258, *p* < 0.001, and partial *η²* 0.078. The contrast analysis between the different strategies revealed that the lexical replacement “route” (*M* = 1.07, SD = 0.42) is significantly better at predicting erroneous replacements of missed consonants than the purely auditory driven strategy (*M* = 1.30, SD = 0.47), *F*(1,133) = 50.813, *p* < 0.001, and partial *η²* 0.276. The comparison between the “lexical” (*M* = 1.07, SD = 0.42) and “frequency” (*M* = 1.03, SD = 0.68) replacement strategies did not yield any significant results (*F*(1,74) = 0.408, *p* = 0.524, and partial *η²* 0.003). This indicates that listeners prefer to replace missed consonants by alternatives that will yield existing words in the target Dutch language instead of phonologically licit but nonsensical alternatives. Yet, at the same time, within the set of possible words, listeners do not opt for the more frequent ones. The mean standardized distances between observed and expected values for erroneous consonant replacement for the auditory, lexical and frequency routes are given in Table 2 and their variation is depicted in Figure 1.

(2) In order to analyze the potential* transfer of phonetic features* of the consonant between stimulus and response, a repeated measures ANOVA was performed comparing the outcomes for voicing, place, and manner of articulation within the lexical replacement strategy only (i.e., the best “model” for the stimulus-response variation resulting from the statistical analysis in (1)). The word-internal position (initial, final) was used as between-subject factor. Test results show that the three articulatory features yield significantly different outcomes (*F*(1.805,240.02) = 12.506, *p* < 0.001, and partial *η²* 0.086). With respect to the between-subject factor, the outcomes for the two possible positions of the consonant within the word (initial versus final) were not significantly different. The mean standardized distances between observed and expected values for erroneous consonant replacement for the speech features “voicing”, “place” and “manner” of articulation are given in Table 3 and their variation is depicted in Figure 2.

Post hoc testing with respect to the three articulatory features indicated that “voicing” is best preserved and that “place” of articulation yields significantly lower, that is, better, scores than “manner” of articulation.

## 7. Discussion

The present study examined to what extent auditory, lexical, and frequency factors may influence erroneous phonemic replacements in an open-set spoken word recognition paradigm. Although the intent of the present study was to verify the presumed auditory nature of consonant replacement strategies in speech audiometric testing, the results of the analysis also address several aspects of human spoken word recognition models.

According to the literature, processing an incoming speech signal may occur in two distinct modes: the* autonomous* mode of processing is characterized by an apparent lack of influence from sources of linguistic knowledge or bias (e.g., the lexicon) whereas the* interactive* mode is actually characterized by the influence of such linguistic knowledge (e.g., a wordhood bias) [44]. Previous research has shown that the processing mode may be determined by the type of speech perception task that is used: whereas recognition tasks (e.g., phoneme or word identification) are expected to show a larger effect of lexical influence, discrimination tasks will rather produce an autonomous mode of processing [45].

Building on these insights the methodology followed in this study may be taken to represent a typical case of the first, that is, the interactive, mode due to the fact that listeners have received the instruction to repeat as much of the words that they have heard. This is essentially a “listen-and-repeat” task that calls for speech recognition at the phonemic and word level. Lexical information is therefore expected to be an important predictor of the speech perception errors that are found in such a listening task. The fact that listeners are encouraged to report anything they hear, even if this yields a nonsense word, may well mitigate this linguistic interaction effect, but it will not be able to eliminate it altogether.

A first important finding of this study is therefore that the erroneous replacement of consonants obtained through speech audiometric testing procedures is the result of combined bottom-up and top-down processes in which linguistic factors take up an important portion. Our results indicate that in the case of word identification lexical information is essentially a better predictor of errors than acoustic similarity.

From the point of view of the audiological practice, spoken word recognition measures are commonly used to describe the extent of a patient's hearing impairment, to make a differential diagnosis of auditory disorders, to determine the needs and type of audiological rehabilitation, to verify benefits of hearing aid use, and to monitor the patient's performance over time for diagnostic or rehabilitation purposes [46]. Within this context, it is therefore important to observe that word recognition errors are highly influenced by nonauditory factors. An identical interpretation of correct responses and erroneous consonant replacements in terms of hearing performance would place disproportionate emphasis on top-down processing strategies and would thus seriously overestimate the proper functioning of the inner ear hearing mechanisms.

Secondly, a more systematic analysis of the reception of the different articulatory features of consonants such as voicing, place, and manner of articulation has shown that in erroneous replacement of consonants “voicing” is best preserved whereas “manner” of articulation is most prone to perception errors. This finding is in line with the literature [7, 8] and with our own expectations: as stated in Section 2, voicing is known to be a perceptually very robust feature. According to the* Auditory Enhancement* hypothesis, its redundant acoustic specifications (presence of low-frequency energy, ratio of consonant duration to preceding vowel duration, and presence/absence of aspiration; see [46]) all work together to make this feature acoustically distinctive even under adverse listening conditions.

## 8. Conclusions

In this study, we have analyzed erroneous phonemic replacements from an open-set spoken word recognition task which is part of a speech audiometric test battery for the Dutch-speaking area. The task was administered to 230 hearing listeners with a native Dutch-speaking background. Erroneous consonant replacements in CVC words were analyzed in terms of the probability of choosing a particular consonant based on acoustic-phonetic, lexical, and frequency variables.

The results indicate that erroneous replacements of consonants are best predicted by lexical information; that is, when part of the incoming speech signal is missing, listeners tend to fill in the gap by picking out one of the stimulus word's phonological neighbors which are part of their mental lexicon. In doing so, listeners do not, however, prefer words that are more frequently heard in the ambient language over alternatives that are less frequent.

Taken together, these results are thought to be of importance for current models of speech perception, pointing in the direction of an interaction between bottom-up and top-down factors even at the lowest levels of spoken word recognition. At the same time, the results are highly relevant for the audiological practice. They draw attention to the fact that erroneous consonant replacements are highly affected by linguistic knowledge. Word repetitions tasks provide a combined auditory-linguistic profile of the patient and might thus not be best suited to measure hearing performance or to guide the rehabilitation of hearing impaired patients. To factor out possible lexical influences on hearing performance measures, they should be complemented by other listening task types that are known to have less linguistic bias, such as phonemic discrimination.

## Figures and Tables

**Table 1:** Database of stimulus-response pairs of Dutch CVC words.

	*N*
Number of participants	230
Total number of word tokens analyzed	21285
Total number of single phoneme errors	1957
Word-initial position	1252
Word-medial position	113
Word-final position	592

**Table 2:** Mean outcomes with standard deviations of the standardized distances between observed and expected values for the auditory, lexical, and frequency routes for each population separately. Post hoc test statistics represent the obtained *p* values after Bonferroni correction for multiple testing.

	Auditory	Lexical	Frequency	Test statistics	
NH	1.30 (SD 0.47)	1.07 (SD 0.42)	1.03 (SD 0.68)	Auditory-lexical Lexical-frequency Auditory-frequency	**p** < **0.001** *p* > 0.05 **p** = **0.001**

**Table 3:** Mean outcomes with standard deviations of the standardized distances between observed and expected values for the speech features “voice,” “place,” and “manner” of articulation for each population separately. Post hoc test statistics represent the obtained *p* values after Bonferroni correction for multiple testing.

	Voice	Place	Manner	Test statistics	
NH	0.28 (SD 0.29)	0.33 (SD 0.28)	0.42 (SD 0.31)	Voice-place Voice-manner Place-manner	*p* = 0.192 **p** < **0.001** **p** = **0.001**

**Table 4:** Two wordlists from [36].

List 17	List 19
Loop	Goud
Fout	Doek
Maai	Hooi
Rood	Sap
Hoek	Lag
Zich	Jong
Dam	Door
Tien	Pijl
Geeuw	Zin
Kok	Bes
Bel	Kieuw
Huis	Neef

**Table 5:** Dutch consonant inventory with articulatory features, according to [37].

Voicing		Place			Manner			
Voiced	Voiceless	Front	Mid	Back	Plosive	Nasal	Fricative	Approximant
b	p	b	d	k	b	m	v	*υ*
d	t	p	t	ŋ	p	n	f	r
v	k	m	n	h	d	ŋ	s	l
z	f	v	z	x	t		z	j
m	s	f	s		k		h	
n	h	*υ*	r				x	
ŋ	x		l					
*υ*			j					
*γ*								
r								
l								
j								
